# Treatment of Mesh Skin Grafted Scars Using a Plasma Skin Regeneration System

**DOI:** 10.1155/2010/874348

**Published:** 2010-07-01

**Authors:** Takamitsu Higashimori, Taro Kono, Hiroyuki Sakurai, Hiroaki Nakazawa, William Frederick Groff

**Affiliations:** ^1^Department of Plastic and Reconstructive Surgery, Tokyo Women's Medical University, Medical Center East, 8-1 Kawada-cho, Shinjuku-ku, Tokyo 162-8666, Japan; ^2^Department of Plastic and Reconstructive Surgery, Tokyo Women's Medical University, 8-1 Kawada-cho, Shinjuku-ku, Tokyo 162-8666, Japan; ^3^Division of Cosmetic Dermatology, La Jolla Cosmetic Surgery Centre, La Jolla, CA 92037, USA

## Abstract

*Objectives*. Several modalities have been advocated to treat traumatic scars, including surgical techniques and laser resurfacing. Recently, a plasma skin regeneration (PSR) system has been investigated. There are no reports on plasma treatment of mesh skin grafted scars. The objective of our study is to evaluate the effectiveness and complications of plasma treatment of mesh skin grafted scars in Asian patients. *Materials and Methods*. Four Asian patients with mesh skin grafted scars were enrolled in the study. The plasma treatments were performed at monthly intervals with PSR, using energy settings of 3 to 4 J. Improvement was determined by patient questionnaires and physician evaluation of digital photographs taken prior to treatment and at 3 months post treatment. The patients were also evaluated for any side effects from the treatment. *Results*. All patients showed more than 50% improvement. The average pain score on a 10-point scale was 6.9 +/− 1.2 SD and all patients tolerated the treatments. Temporary, localized hypopigmentation was observed in two patients. Hyperpigmentation and worsening of scarring were not observed. *Conclusions*. Plasma treatment is clinically effective and is associated with minimal complications when used to treat mesh skin grafted scars in Asian patients.

## 1. Introduction

Laser treatment of scars was first reported in 1980s using continuous wave carbon dioxide (CO_2_), argon and neodymium: yttrium–aluminum garnet(YAG) lasers. Results were operator dependent and scar recurrence or worsening of scarring was observed [1]. In the 1990s, high energy pulsed CO_2_ and erbium:YAG (Er:YAG) lasers had become available and did improve scars and caused fewer significant side effects [2]. However, the epidermis is significantly damaged with use of these lasers and this can result in potential adverse effects, including prolonged erythema and pigmentary disturbances, especially in Asians [3].

The plasma skin regeneration system (PSR; Portrait, Rhytec, Inc., Waltham, MA) is a novel device that utilizes radiofrequency to convert nitrogen gas into a high energy state of matter called plasma. PSR has been shown to remove benign skin lesions with similar efficacy and a lower complication rate when compared to the CO_2_ laser [4]. We first reported the effectiveness of plasma treatment in improving traumatic scarring in 2009 [5].We hypothesized that the PSR system would be effective in improving mesh skin grafted scars, with minimal downtime and few adverse effects.

## 2. Materials and Methods

Four Asian patients with traumatic scars and Fitzpatrick skin types III or IV were enrolled in the study (Table 1). Patients with a history of keloid formation, prior dermabrasion or laser treatment, or isotretinoin use within the last 6 months were not enrolled in the study. The plasma treatments were performed at monthly intervals, using energy settings of 3 to 4 J (15 to 20 msec), a single pass and no overlap. Before each plasma treatment, seven percent lidocaine cream was applied to the treatment site for 1 hour. Immediately after treatment, patients were asked to rate their pain level. Pain was assessed using a visual analog scale (VAS, 0–10). Patients were seen 1 week after each treatment and also 3 months after the last treatment. Blinded assessments of the treatment response were made by 2 expert plastic surgeons who examined digital photographs taken pretreatment (baseline) and 3 months after the last treatment. Apparent improvement of the lesion was given a score based on the following: 0 = 0%; 1 = 1% to 25%; 2 = 26% to 50%; 3 = 51% to 75%; and 4 = 76% to 100% improvement. Adverse effects, including pigmentary changes and worsening of the traumatic scarring, were recorded by the same investigator who performed the treatments. Case 2 and Case 4 completed only one and three treatments, respectively, because they were satisfied with their results. 

## 3. Results

**Figure 1:**
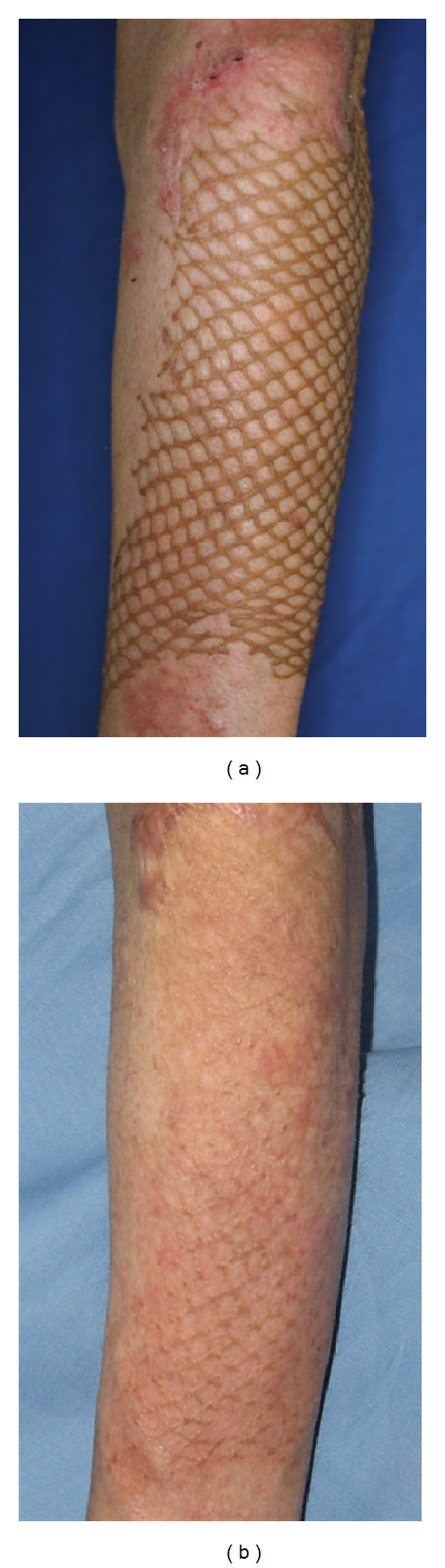
Twenty eight-year-old male, mesh skin grafted scars on left forearm (deep burn caused by explosion, age of scar was 2 months) before (a) and after eight treatment (b). Significant improvement was observed.

**Figure 2:**
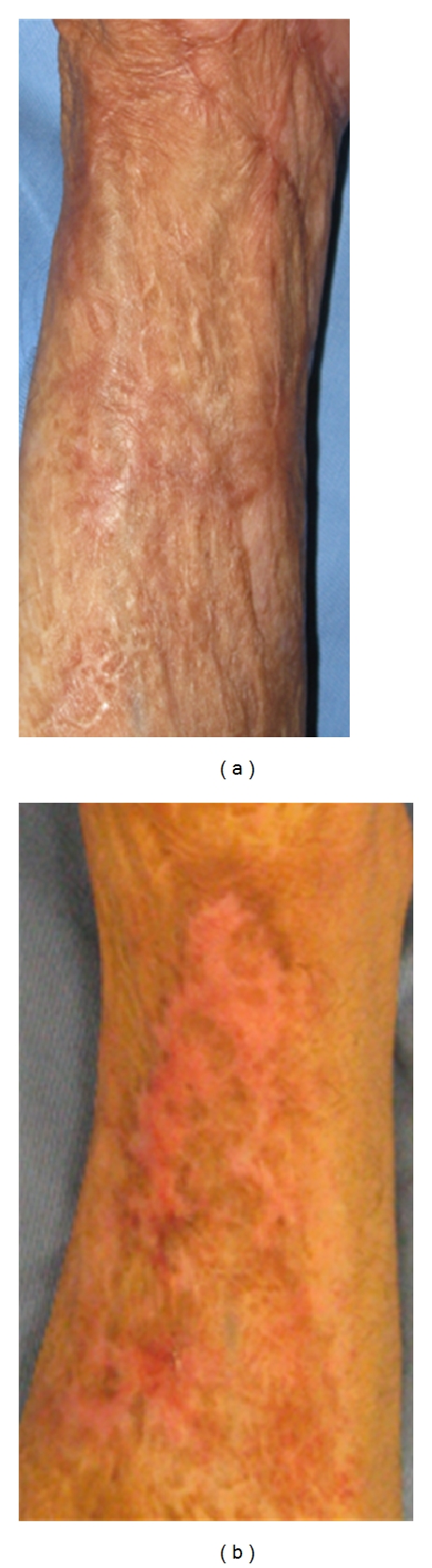
Fifty three-year-old male, mesh skin grafted scars on left forearm (deep flame burn caused by accident, age of scar was 17 months) before (a) and after one treatments (b). Moderate improvement was observed but hypopigmentation was occurred and gradually improved within several months.

**Figure 3:**
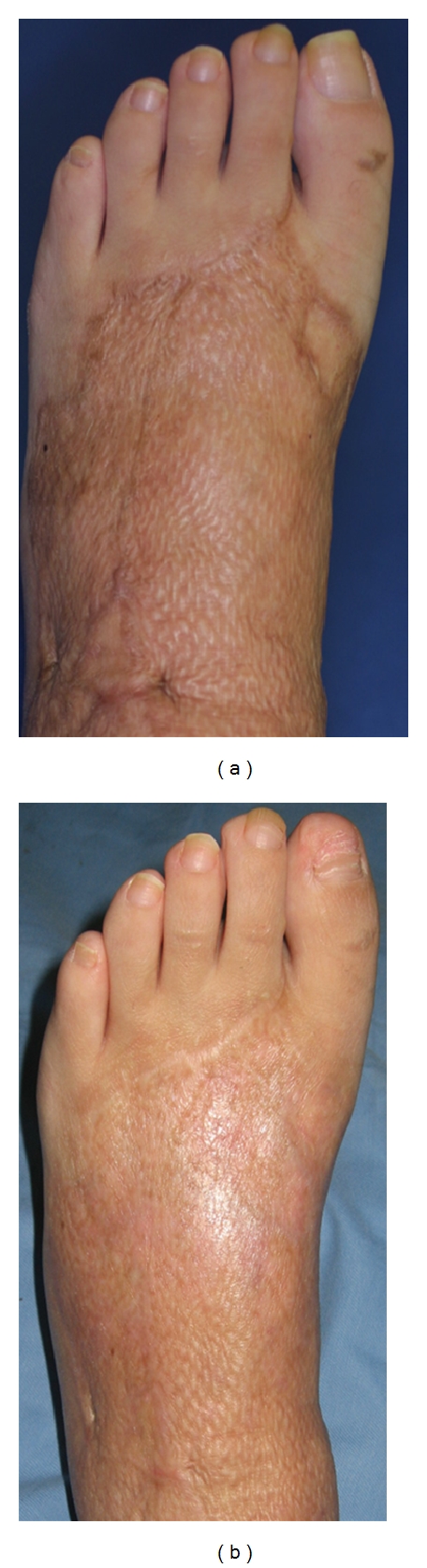
Thirty four-year-old female, mesh skin grafted scars on left foot (deep burn caused by explosion, age of scar was 11 months) before (a) and after eight treatments (b). Significant improvement was observed.

**Figure 4:**
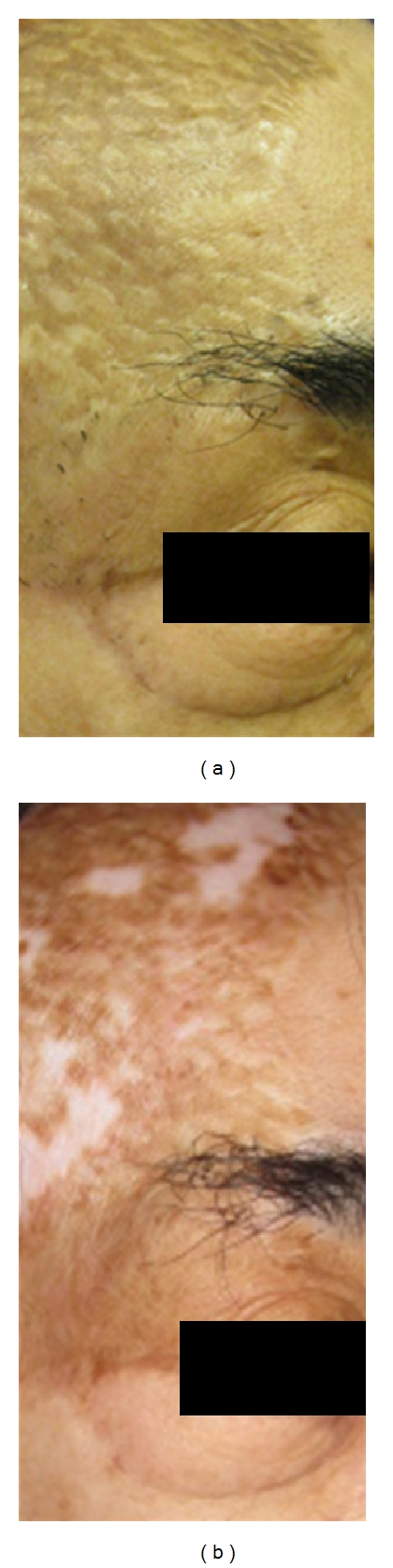
Fifty one-year-old female, mesh skin grafted scars on left foot (deep burn caused by explosion, age of scar was 11 months) before (a) and after eight treatments (b). Moderate improvement was observed but hypopigmentation was occurred.

All patients completed the study (Figures 1, 2, 3, and 4). Case 1 and 3 experienced significant improvement. Case 2 and 4 experienced moderate improvement (Table 2). The average pain score on a 10-point scale was 6.9 +/− 1.2SD and all patients tolerated the procedures. The epithelization time after treatment was 14.7 ± 7.3 days (average ± SD). Unfortunately, Case 2 could not come to our clinic because they had moved away, therefore we could not follow them regularly. Temporary and local hypopigmentation was observed in two cases and the hypopigmentation in Case 4 gradually improved within 6 months. Hyperpigmentation and worsening of scarring were not observed as a result of the procedures (Table 3).

## 4. Discussion

While dramatic improvement is seen when using ablative CO_2_ laser resurfacing to treat facial traumatic scars, it is operator dependent. 

Moreover, the epidermis is significantly damaged during this process and this can be associated with serious adverse effects, particularly when using this modality to treat scarring of the face. These side effects include transient or prolonged erythema, temporary and permanent hypopigmentation, hyperpigmentation, infection, and scarring, especially in Asians [1, 3]. Hædersdal et al. reported that the nonablative 1540 nm fractional laser improved the texture of mature burn scars, particularly in superficial burn scars [6]. 

The plasma skin regeneration system is a novel device that utilizes radiofrequency to convert nitrogen gas into a high energy state of matter called plasma [7–9]. The plasma is emitted at 5–15 millisecond pulses into the skin and can be delivered at 1–4 J of energy. Because the plasma skin regeneration device is not a laser, its mechanism of action is not dependent on chromophores. There is no direct means for radiofrequency transfer into the treatment area. Rapid heating of the skin occurs as the excited gas gives up energy to the skin. The epidermis becomes nonviable and there is controlled thermal modification to the underlying dermis with minimal thermal injury to surrounding tissues. The zone of thermal injury is less than the CO_2_ laser [4].

Plasma treatment is used for skin rejuvenation. Bogle et al. reported that with plasma skin rejuvenation, using a multiple treatment, low-energy (1.2–1.8 J) technique, physicians can successfully improve photodamaged facial skin with minimal downtime [7]. In Bogle's study, re-epithelialization was complete in 4 days and only temporary hyperpigmentation was observed. 

More recently, Gonzalez et al. reported a single high-energy (3.5–4 J) plasma treatment can significantly improve acne scarring [10]. Their study demonstrates that, while the magnitude of improvement with plasma treatment is not as dramatic as that seen with multipass CO_2_ laser treatment, a high energy, double pass, PSR treatment requires minimal operator training to achieve predictable and safe improvement in acne scars. 

In contrast to aggressive ablative techniques, plasma regeneration maintains the integrity of the epidermis, which leads to less social downtime and less risk of significant side effects. 

In our study, plasma treatment was effective in improving mesh skin grafted scars in Asian with minimal side effects. 

We reported previously that the PSR is not effective for deep scars because good remodeling could not be achieved, so wide and deep scars, such as abdominal surgery scars, are not good candidates for plasma treatment [5].

Hyperpigmented scars were improved in color after plasma treatment and the risk of adverse effects was minimal, even in dark skin patients [5]. In this study, one patient developed hypopigmentation after plasma treatment with 4 J. This improved over a 6-month period.

In contrast to the CO_2_ laser, plasma treatment maintains the integrity of the epidermis, leading to low risk of scar formation. Plasma treatment appears to provide a safe and effective treatment of mesh skin grafted scars.

## 5. Conclusion

In this study, plasma treatment appears to be safe and effective for treating mesh skin grafted scars. However, hypopigmentation is a potential complication of this treatment. Future studies with a larger patient size are needed to evaluate the safety and effectiveness of this procedure. 

## Figures and Tables

**Table 1:** Patients list.

Case	Age	Sex	Region	1st treatment from skin grafting	Treatment times
1	28	male	forearm	2 months	8
2	53	male	forearm	6 months	1
3	34	female	leg	11 months	8
4	51	male	forehead	17 months	3

**Table 2:** Degree of improvement (*n* = 4).

Worse	Poor, 0–25%	Mild, 26–50%	Moderate, 51–75%	Significant, 76–100%
0/4	0/4	0/4	2/4	2/4

**Table 3:** Complications (*n* = 4).

VAS pain score (0–10)	6.9 ± 1.2
Epithelization time (days) after plasma treatment	14.7 ± 7.3
Infections	0/4
Hyperpigmentation	0/4
Hypopigmentation	2/4
Worsening of scarring	0/4
